# A two-dimensional conceptual framework for understanding mental well-being

**DOI:** 10.1371/journal.pone.0214045

**Published:** 2019-03-27

**Authors:** Mohsen Joshanloo, Dan Weijers

**Affiliations:** 1 Department of Psychology, Keimyung University, Daegu, South Korea; 2 Philosophy Programme, University of Waikato, Hamilton, New Zealand; College of William and Mary, UNITED STATES

## Abstract

The complex nature of mental well-being is reflected in the great diversity of variables thought to represent aspects of mental flourishing. Discovering the underlying structure of mental well-being is important for a full understanding of this complex construct. Using data from 3 countries (the United States, Japan, and Iran), we performed multi-dimensional scaling to analyze the representation of 9 hedonic and eudaimonic well-being variables in a 2-dimensional psychological space. The analyses revealed 2 interpretable underlying dimensions across cultures and gender groups. The first dimension—eudaimonic well-being versus hedonic well-being—is well-known to well-being researchers. The second dimension—existential relatedness versus Epicurean independence—has not been recognized before. Existential relatedness is the characteristic of being meaningfully interconnected with things other than oneself, and is mainly based on the variables positive relations with others, personal growth, purpose in life, and life satisfaction. Epicurean independence is the characteristic of being relatively free of painful experiences and not feeling the need to have ones’ views accepted by anyone but oneself. Epicurean independence is mainly based on the variables autonomy and absence of negative affect. We explain these dimensions in detail and discuss the implications for well-being research and policy.

## Introduction

Researchers have developed a large number of mental well-being variables. This rich diversity of mental well-being variables is regarded as necessary and useful, reflecting the complex nature of mental well-being itself [1]. However, psychological concepts tend to cluster together under broader or higher-order constructs that should also be investigated for a complete understanding of these variables. In other words, observing the relationships between these variables, and the underlying patterns of the relationships, will enable researchers to better understand the nature of mental well-being [2]. Hence, another important step in analyzing the concept of well-being is to examine the underlying structure of mental well-being variables.

To understand the underlying structure of mental well-being variables, one strategy could be to inspect their correlation matrixes. However, a relatively large number of variables seldom produce patterns of correlations that are clear and easy to discern from correlation matrices. As Fabrigar and Wegener [3] point out, this makes it “difficult to gauge whether an observed pattern of correlations is sufficiently close to a hypothesized pattern to support the appropriateness of a particular structural representation of the data (e.g., all the measures assessing one construct or a certain small set of constructs)” (p. 3). Consequently, researchers have turned to factor analysis to identify the unobservable concepts (i.e., factors) that can account for the pattern of correlations among mental well-being variables [4–7]. Factor analysis is a powerful data reduction tool that provides useful information about the nature of the factors, the magnitude and direction of relationships between the factors, and the magnitude and direction of the factors’ influences on observed variables (i.e., factor loadings). Such information offers important contributions to our understanding of the nature of mental well-being.

Yet, factor analysis suffers from some inherent limitations. For example, in simple-structure Confirmatory Factor Analysis (CFA) each mental well-being variable is allowed to load on only a single latent factor, while its loadings on other latent variables are constrained to zero. It is increasingly recognized that with real data, cross-loadings are far from rare [8]. Previous research shows that, particularly in the measurement model of mental well-being, zero secondary loadings are rare [9]. Hence, constraining secondary loadings to zero may be unrealistic in many circumstances, resulting in biased estimates [10]. Moreover, in CFA, the relationships between an indicator and non-target factors, and the relationships between all of the indicators, are usually fixed at zero, which means these relationships may go unnoticed. Many structural equation modelling computer tools output predicted correlations (and/or covariances) between all variables of the model [11]. Yet, with a relatively large number of variables, the resulting matrices are not much more useful than bivariate correlation matrices in discerning underlying dimensions. Moreover, in practice, model-implied correlation matrices are rarely independently explored in order to uncover underlying structures in CFA studies.

Classic Exploratory Factor Analysis (EFA) [3] and a more recent and powerful related technique called Exploratory Structural Equation Modelling (ESEM) [10] provide additional information on the relationship between indicators and non-target factors by freely estimating all of the non-target factor loadings [9, 12, 13, 14]. This allows for the relationship between each variable and all of the factors to be determined, which is a clear advantage over CFA for these exploratory techniques. However, in these techniques too, researchers usually do not probe all pairwise relationships between all of the observed variables in pursuit of underlying dimensions and patterns. This may be caused by the difficulties associated with discerning meaningful patterns from the complex correlation matrices that these techniques produce.

Despite these limitations, factor analytic findings have provided undeniably important insights into the structure of mental well-being variables, without which further progress would not have been possible in this field of research. In particular, a general finding that stands out from previous factor analytic studies is that mental well-being is a multi-dimensional rather than a unidimensional construct [13]. The value of such robust findings is, for example, to help protect the field against reductionist approaches that would reduce mental well-being to a single dimension, resulting in the cost of losing valuable information about this complex construct. In sum, factor analysis has certain strengths, and it has been tremendously useful for the field of mental well-being, yet it can be supplemented by other exploratory data reduction techniques that impose less constraints on the parameter estimates.

Unfortunately, researchers have largely ignored other techniques to systematically organize and categorize mental well-being variables. In the present study, we used a statistical technique called Multidimensional Scaling (MDS) on mental well-being data in order to identify the underlying structure of mental well-being. Exploratory MDS is free from some of the statistical constraints of factor analysis, allowing the data to more freely speak for themselves. MDS also enables simple and effective data visualization, which enables more holistic and intuitive elucidation of the pairwise relationships between the variables and data’s underlying dimensions. Therefore, we believe that MDS has much to offer to the field of mental well-being. Below, we describe MDS and its potential for clarifying the structure of mental well-being variables in more detail.

### MDS and its merits

Large complex matrices of intercorrelations are often difficult to holistically explore and interpret. MDS is a data reduction technique that provides a visual representation of the empirical intercorrelations in order to facilitate the exploration and interpretation of the data. MDS outputs a spatial map conveying the relationships among variables, wherein similar variables are placed closer and dissimilar variables are placed further apart from each other. Such maps enable researchers to actually “see” the empirical data, potentially providing unique insights into the otherwise complex matrix of associations among variables [15, 16]. The highly intuitive visual outputs of MDS are utilized to identify underlying dimensions that reflect the structure of complex psychological phenomena. These dimensions are straight lines (i.e., principal axes) along which the variables are scattered in a typically 2-dimensional space. Dimensions need to be interpreted by the researcher in correspondence with prior knowledge about the variables. Interpretation is facilitated by identifying the variables located at the two ends of each dimension [17].

Although MDS can be used in confirmatory ways, it is best used as an exploratory data reduction technique, characterized by flexibility and relative freedom from strict theoretical boundaries [17, 18]). Therefore, this technique is particularly helpful when the organization of concepts and hidden structures are relatively unknown. In many fields of inquiry, existing theory is “often insufficiently precise for [the purpose of] specifying hypotheses to the degree of precision required by confirmatory analyses, hence the need for exploration” [19]. This particularly applies to the field of mental well-being, within which research on the dimensionality of mental well-being has almost exclusively relied on factor analysis. The near total reliance on a single data analytic technique when promising alternatives are available could lead to missing psychologically meaningful dimensions along which mental well-being variables vary. As a result, there may be hidden structures in mental well-being data awaiting discovery. In sum, exploratory MDS can offer illuminating insights into the nature and structure of mental well-being, while avoiding *a priori* theoretical commitments.

### The present study

This study uses MDS to identify the dimensions along which nine important well-being variables vary within three cultures. The three cultures include the United States of America (USA), Japan, and Iran, which differ in various cultural, religious, political, and socio-economic characteristics. For example, Japan and the USA possess remarkably higher economic development than Iran [20], Iran and Japan are less individualistic than the USA [21], and Iran is remarkably higher in religiosity than the other two nations [22]. This cultural diversity provides a favorable opportunity to examine the potential cultural similarities and differences in any mental well-being dimensions emerging from MDS.

The mental well-being dimensions used in this study include positive affect, the absence of negative affect, and life satisfaction. These three are the most commonly used variables to conceptualize subjective/hedonic well-being [23]. What these three variables share is their emotional and subjective nature as opposed to other more objective and functional mental well-being variables. These variables generally measure how people feel and think about their lives. In factor analytic studies on mental well-being variables, these three variables tend to form a separate latent factor [9].

Other included variables were the six elements of Ryff’s psychological well-being model [24]: autonomy, environmental mastery, personal growth, positive relations, purpose in life, and self-acceptance. These variables capture the points of convergence among the literatures from developmental, clinical, existential, and personality psychology on the optimal functioning of humans [25]. The basic difference between psychological well-being and subjective well-being is that psychological well-being predominantly captures various domains of functioning rather than feeling. Ryff’s model of psychological well-being is recognized as the first, and one of the most widely used, empirically based models of eudaimonic or functional well-being [26]. In factor analytic studies of subjective and psychological well-being variables, the six psychological variables tend to form a separate latent factor, with some cross-loadings [9].

Although not exhaustive, we believe that these nine subjective and psychological well-being variables adequately represent much of the vast diversity in mental well-being variables as recognized by psychologists and philosophers. The nine variables have gained increasing popularity in the social sciences, and have been extensively used to measure mental well-being across cultures [26]. Using MDS as an exploratory analytical tool, the present study aimed at uncovering hidden structures underlying this rather comprehensive array of mental well-being variables across three diverse cultural groups.

This study also sought to look at the gender differences in the facets and dimensions of well-being. Not much is known about gender differences in the underlying structure of well-being variables. Yet, a recent study by Joshanloo [27] using a short well-being scale in a large sample from the USA provided some preliminary evidence in favor of gender similarity. The study uncovered highly similar general structures for well-being across gender. Similarly, previous research using multiple-group CFA to examine the measurement invariance of well-being scales shows that there are many more similarities between genders than differences in the factor structures of well-being across cultures [28–30]. Research comparing the levels of well-being dimensions across genders has also generally demonstrated small differences or the absence of gender differences [31]. Given the findings demonstrating a large degree of gender similarity in the levels and factor structure of well-being, we expected largely similar structures to emerge across genders.

## Methods

### Participants

#### USA

This study used the data produced during 2004–2006 in the second wave of the National Study of Midlife in the United States (MIDUS 2) [32]. The overall MIDUS 2 sample consists of 4963 respondents. However, 922 participants had missing values on all nine variables of the study, and 53 participants had between 1 to 8 missing values. We only used 3988 participants (55.4% females) who had no missing data on the nine variables of the study (*M*_age_ = 56.12, *SD*_age_ = 12.34). More information about data collection procedures can be found at http://midus.wisc.edu.

#### Japan

This study used the data produced during 2012 in the second wave of the Survey of Midlife Development in Japan (MIDJA 2) [33]. The overall MIDJA 2 sample consists of 657 respondents. However, 22 participants had between 1 to 6 missing values. Our final sample consisted of 634 participants (52.4% females) who had no missing data on the nine variables of the study (*M*_age_ = 58.89, *SD*_age_ = 13.41). The MIDJA measures parallel those in the MIDUS. The scales of the study were translated into Japanese using the method of back-translation. More information on the survey can be found at http://midus.wisc.edu/midja.

#### Iran

We used a convenience sample of 527 university students studying at universities in Tehran. However, we excluded 2 participants that had missing values on some of the nine variables, leaving a final sample of 525 (58.1% females, *M*_age_ = 21.70, *SD*_age_ = 2.68). The study was voluntary, anonymous, and in Persian. Informed consents were obtained from the participants. The scales of the study were translated into Persian using the method of back-translation.

The American and Japanese data were collected in national projects and their methods and procedures are described on the MIDUS/MIDJA website. Data collection in Iran was supervised by the first author in conformity with all international research and ethical standards. However, IRB approval was not required for psychological surveys in the data collection sites in Iran at the time of data collection. Inquiries concerning the Iranian dataset may be directed to the first author. The Iranian data file is supplied as supporting information (S1 Dataset).

### Measures

#### Affect

The 12-item negative and positive affect scale [34, 35] was used to measure positive and negative affect in all three countries. Respondents indicate how often (from 1 = *all* to *5 = none of the time*) during the past 30 days, they felt six positive and six negative affective states. All the responses are recoded such that higher scores indicate higher frequency of the experienced emotions. In all the analyses, negative affect was reverse-coded to represent the absence of negative affect.

#### Life satisfaction

Life satisfaction was assessed in the USA using the 5-item MIDUS life satisfaction scale. The items measure satisfaction with overall life, work, health, relationship with spouse/partner, and relationship with children. Each item is coded from *the worst possible* (0) to *the best possible* (10). In Iran, the Satisfaction With Life Scale (SWLS) [36] was used to measure life satisfaction. Each of the five items of this measure is rated on a 7-point scale ranging from *strongly disagree* (1) to *strongly agree* (7). In Japan, both the MIDUS life satisfaction scale and the SWLS were included. Therefore, two separate analyses were conducted for this country, to examine the impact of scale use on the general structure of the variables.

#### Psychological well-being

In the USA and Japan, a 42-item version of Ryff’s psychological well-being scales [24] was used to measure the six elements of psychological well-being. In Iran, the 54-item version [24] of the scale was used. Items are scored on a 7-point scale ranging from *strongly disagree* (1) to *strongly agree* (7).

### Statistical analysis

MDS represents proximity data (e.g., measures of similarity/dissimilarity or correlation) as distances among points in a typically 2-dimensional space. Beginning with some starting configuration, the scaling moves iteratively, so that the fit between distances and data is improved until no further improvement seems possible in the resulting geometric space. The data in the present study were analyzed by means of the MDS technique PROXSCAL [37] in SPSS 25. PROXSCAL is recognized as one of the most technically up-to-date, and most widely used and recommended MDS algorithms [15]. PROXSCAL offers several improvements over other scaling algorithms (e.g., ALSCAL), including algorithmic strategies for ensuring better convergence [38]. As is typical in modern applications of MDS, the analysis was based on Euclidean distances and Z transformation initialized with the Torgerson solution [15, 39]. Given the scales of the measured variables, an interval proximity transformation was used. We chose a 2-dimensional solution for the present data for several reasons: To achieve greater parsimony, to increase the chances of the findings being replicated in future research, to increase the generalizability of the findings to other national samples, to facilitate more effective interpretation and communication of the results [15, 17], and because nine variables may be insufficient to reliably infer more than two dimensions. Model-data fit was assessed in terms of Kruskal's Stress-1, with a value of 0 indicating a perfect MDS solution. Stress-1 values greater than .20 are considered to indicate unacceptable fit [40].

## Results

**Fig 1:**
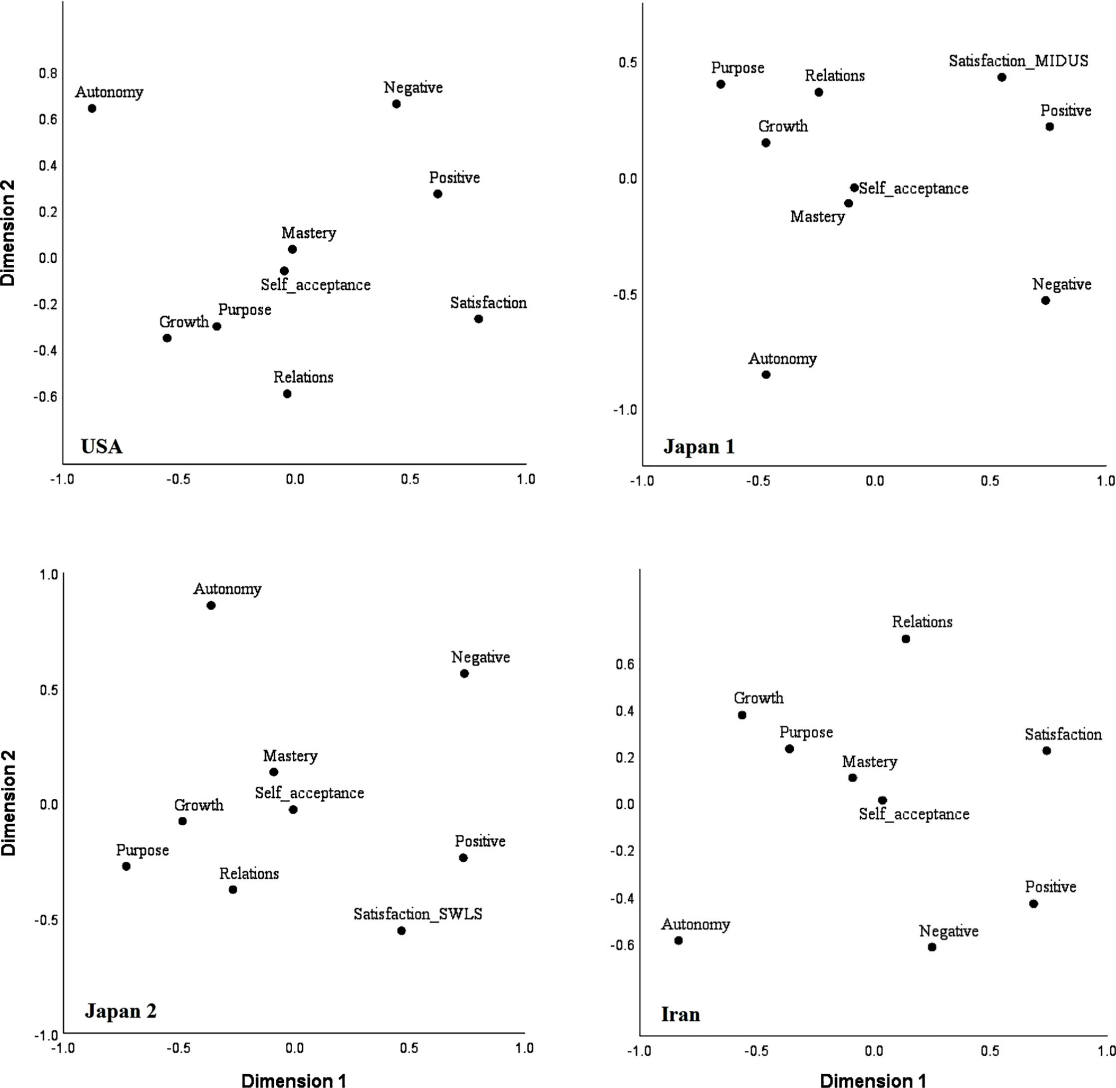
Two-dimensional MDS plots across nations. Dimension 1: hedonic well-being vs eudaimonic well-being. Dimension 2: existential relatedness versus Epicurean independence. Japan 1 = Japanese analysis using the MIDUS life satisfaction scale. Japan 2 = Japanese analysis with the SWLS.

The internal consistencies and correlations between the variables are presented in Table 1. Fig 1 displays the 2-dimensional MDS plots for the three countries. The Stress-1 values were smaller than the cutoff point of .20 in the USA (.11), Japan with the MIDUS life satisfaction scale (.11), Japan with the SWLS (.13), and Iran (.14), indicating acceptable fit for the 2-dimensional solution in all countries. The MDS solutions displayed in the plots are similar, with a few minor differences. In particular, there is a high level of consistency with respect to the variables located at the opposite ends of each of the dimensions (which are usually afforded more weight in dimension interpretation [17]). On the horizontal dimension in the present three solutions, the three subjective well-being variables have clearly clustered on one side, and psychological well-being variables have clustered on the other side. Thus we may infer a primary dimension of eudaimonic well-being versus hedonic well-being. This result is in line with Joshanloo’s MDS analysis of a short well-being scale, which found robust evidence of a hedonic vs eudaimonic dimension [27].

**Table 1:** Internal consistencies and correlations.

		α	1	2	3	4	5	6	7	8
USA										
	1.Positive affect	.90	1.00							
	2.Negative affect (absence)	.85	.61	1.00						
	3.Life satisfaction (MIDUS)	.65	.56	.50	1.00					
	4.Autonomy	.71	.30	.32	.23	1.00				
	5.Environmental mastery	.78	.58	.58	.55	.52	1.00			
	6.Personal growth	.75	.41	.38	.39	.43	.58	1.00		
	7.Positive relations	.78	.46	.39	.45	.38	.62	.58	1.00	
	8.Purpose in life	.70	.44	.44	.44	.40	.63	.69	.60	1.00
	9.Self-acceptance	.84	.60	.54	.56	.51	.76	.64	.66	.68
Japan										
	1.Positive affect	.93	1.00							
	2.Negative affect (absence)	.86	.46	1.00						
	3.Life satisfaction (MIDUS)	.72	.61	.46	1.00					
	4.Autonomy	.73	.23	.27	.24	1.00				
	5.Environmental mastery	.75	.47	.48	.53	.54	1.00			
	6.Personal growth	.80	.34	.32	.42	.41	.63	1.00		
	7.Positive relations	.78	.43	.34	.44	.33	.61	.66	1.00	
	8.Purpose in life	.58	.34	.25	.36	.32	.54	.66	.56	1.00
	9.Self-acceptance	.79	.52	.45	.54	.53	.72	.66	.63	.52
Japan										
	1.Positive affect	.93	1.00							
	2.Negative affect (absence)	.86	.46	1.00						
	3.Life satisfaction (SWLS)	.90	.59	.33	1.00					
	4.Autonomy	.73	.23	.27	.28	1.00				
	5.Environmental mastery	.75	.47	.48	.50	.54	1.00			
	6.Personal growth	.80	.34	.32	.38	.41	.63	1.00		
	7.Positive relations	.78	.43	.34	.44	.33	.61	.66	1.00	
	8.Purpose in life	.58	.34	.25	.32	.32	.54	.66	.56	1.00
	9.Self-acceptance	.79	.52	.45	.64	.53	.72	.66	.63	.52
Iran										
	1.Positive affect	.88	1.00							
	2.Negative affect (absence)	.82	.60	1.00						
	3.Life satisfaction (SWLS)	.87	.50	.41	1.00					
	4.Autonomy	.69	.21	.33	.22	1.00				
	5.Environmental mastery	.73	.49	.51	.53	.46	1.00			
	6.Personal growth	.70	.30	.37	.34	.38	.60	1.00		
	7.Positive relations	.82	.41	.42	.41	.30	.58	.46	1.00	
	8.Purpose in life	.74	.36	.48	.42	.39	.70	.71	.51	1.00
	9.Self-acceptance	.83	.56	.56	.63	.52	.73	.58	.57	.65

*Note*. All correlations coefficients were significant at *p* < .01. Alphas are from the MIDUS and MIDJA documentations of scales. SWLS = Satisfaction With Life Scale.

**Fig 2:**
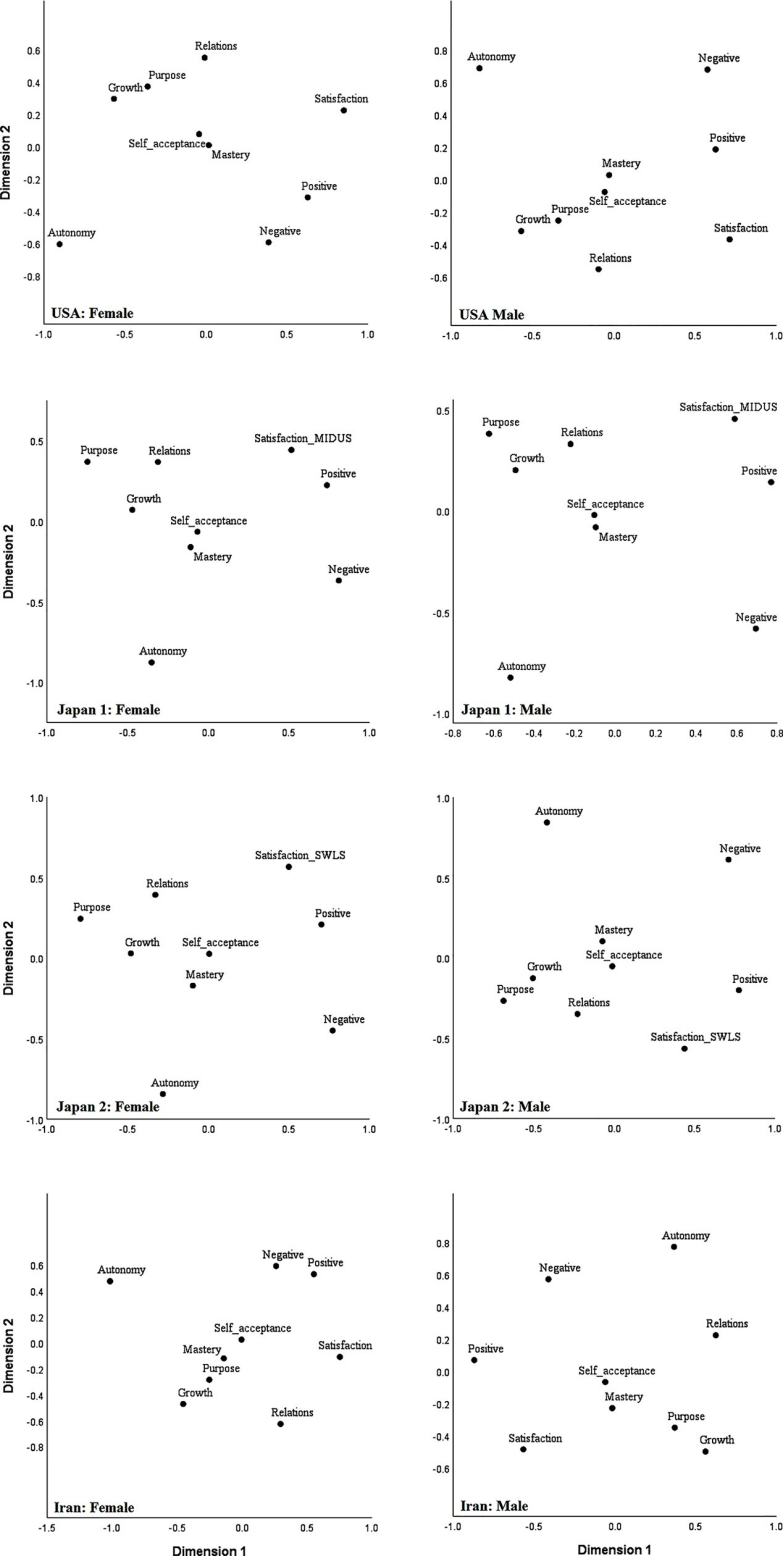
Two-dimensional MDS plots across national and gender groups. Dimension 1: hedonic well-being vs eudaimonic well-being. Dimension 2: existential relatedness versus Epicurean independence. Japan 1 = Japanese analysis using the MIDUS life satisfaction scale. Japan 2 = Japanese analysis with the SWLS.

Concerning the vertical dimension, inspection of the relative positions of the variables across the 2-dimensional space indicates that purpose in life, personal growth, positive relations, and life satisfaction have clustered at one side, a region that can be titled “existential relatedness”, given the existential and relational contents of these variables. Autonomy and the absence of negative affect, on the other hand, have clustered at the opposite side of the solutions, which may be collectively titled “Epicurean independence”. The primary motivation for this title is the view, initially articulated and popularized by Epicurus [41], that the absence of negative emotions is the key to well-being. Although minor differences in the placement of individual variables along this dimension are noticeable across the nations, the general placement of the variables at the opposite ends of the dimension is quite consistent. Hence, we may infer a second dimension of existential relatedness versus Epicurean independence. These dimensions are thoroughly discussed in the next section. Fig 2 displays the MDS plots across gender and nation. Again, despite some minor differences, the same two underlying dimensions can be clearly identified across gender groups in the three nations. Thus, the two general dimensions seem to be applicable to the national and gender subgroups.

Perhaps the only structurally important difference that emerged between the national MDS solutions is the position of positive affect on the vertical dimension. In the USA and Iran, positive affect is closer to the Epicurean independence end of the dimension, whereas in the Japanese solution, positive affect is closer to the existential relatedness end of the dimension. To accommodate for this cultural difference, and to infer more universally applicable dimensions, we did not weight positive affect in this dimension’s interpretation and labeling. However, considering the highly consistent meta-structure emerging across the three countries, this lack of correspondence in the location of a single variable does not seem to be highly consequential. When comparing the MDS solutions across groups, the stability of the meta-structure of the MDS solutions matters more than simple point-by-point correspondences [15], and in our results the stability of the general structural configuration across the national and gender groups is clear.

## Discussion

The purpose of using MDS in this study was to use the graphical outputs to identify underlying dimensions and concepts in mental well-being data, including dimensions that may have been overlooked by previous research. A specific advantage of MDS over the other approaches is that its graphical outputs allow for one-to-many and many-to-many relationships among the variables to be automatically and conveniently observed. In this way, MDS proved to be useful. The graphical output demonstrated two dimensions: the well-known eudaimonic well-being versus hedonic well-being dimension, and the existential relatedness versus Epicurean independence dimension. Demonstrating the value of the graphical outputs, neither of these dimensions are easily identifiable from the intercorrelation matrix shown in Table 1. In particular, the emergence of the existential relatedness versus Epicurean independence dimension from the data can be considered a unique contribution of the present analysis.

### Dimension 1: The eudaimonic versus hedonic dimension

The eudaimonic well-being versus hedonic well-being dimension (Dimension 1) comes through clearly in Figs 1 and 2. Aligning with previous research using a variety of methods [9, 27, 42, 43, 44], the study variables appeared to fall into two groups along the horizontal axis, with Ryff’s eudaimonic variables predominantly in one group and the affect and satisfaction variables, usually associated with hedonic well-being, in the other group.

The variables autonomy, personal growth, purpose in life, and to a lesser extent positive relations with others, self-acceptance, and environmental mastery, constitute the eudaimonic pole of Dimension 1. The clustering of these eudaimonic well-being variables on one side of Dimension 1 suggests that judging oneself by one’s own standards, feelings of self-improvement over time, having meaningful goals in life, and to a lesser extent enjoying good relationships with others, accepting oneself, and being able to fruitfully use features of the external world tend to co-vary together, and in ways that the hedonic well-being variables do not. Eudaimonic well-being was originally an Aristotelian idea, and is best captured by the notion that what makes our lives go well for us is the exercise of excellent character traits [45]. Aristotle himself explained his eudaimonic account of well-being by contrasting it to hedonic accounts of well-being. Rather than viewing the good life as a continuous state of mind, such as constantly feeling pleasure, Aristotle argued that achieving the good life is an active process–actually doing things, not just feeling things [45]. An appropriate translation for eudaimonia is “flourishing”, which captures Aristotle’s concept of well-being because his philosophy was grounded in naturalism, and his view of the good life was wanting for nothing because you were all you could be *given your natural kind* [45–49]. As such, understanding eudaimonia as it was originally intended, and not as the more hedonically flavored “happiness”, as it is sometimes translated as, helps support the labeling of eudaimonic well-being and hedonic well-being on opposite ends of the spectrum that is Dimension 1.

The hedonic well-being pole of Dimension 1 constitutes positive affect, the absence of negative affect, and life satisfaction. In other words, feeling good, not feeling bad, and being satisfied with life tend to co-vary with each other, and not necessarily or nearly as closely with the eudaimonic well-being variables. There is already a great deal of empirical evidence supporting the close relationship between these hedonic well-being variables [9]. And, although it is now understood that there are empirical and conceptual differences between these hedonic well-being variables [50], many philosophers of the past saw them as either intimately related or simply identical in all but name[51]. Notably, the most famous proponent of hedonism, [52], argued that pleasure and satisfaction (along with many cognate terms) were all names for the underlying concept of positive experience. So for Bentham, and many other philosophers from his era, being happy was feeling good, which either meant or entailed also not feeling bad and being satisfied.

Of interest to cross-cultural psychologists, the visual output in Fig 1 shows that the three hedonic well-being variables group together at about the same distance from the eudaimonic variables in all of the countries, despite the considerable cultural differences between the samples. Cross-cultural research on hedonic well-being has demonstrated cultural differences in the extent to which positive and negative affect co-vary with life satisfaction [50, 53]. Despite likely cultural differences in the relationships between the hedonic well-being variables, they group in a very similar way when viewed on the eudaimonic well-being versus hedonic well-being dimension. This suggests two things for the three cultures included in this study. First, that the underlying eudaimonic-hedonic well-being dimension is cross-culturally robust. And second, that any differences in the relationships between the hedonic well-being variables across cultures pales in comparison to the difference between eudaimonic and hedonic variables.

A striking feature of the eudaimonic well-being versus hedonic well-being dimension is that it can be observed in every panel of the figures; it appears in the data for all three cultures and in both male and female subgroups for each culture. So, despite the deep cultural differences between the highly religious Iranian sample, the wealthy and collectivist Japanese sample, and the wealthy and individualistic sample from the United States [20–22], the data support the existence of a robust eudaimonic well-being versus hedonic well-being dimension.

Despite aligning with the vast majority of research that supports the eudaimonic well-being versus hedonic well-being distinction, finding this distinction using MDS is an important advance in happiness studies. Kashdan, Biswas-Diener, and King [54] started a major debate by arguing that distinguishing between eudaimonic and hedonic well-being has its disadvantages, and is not necessarily supported by existing studies. Kashdan and colleagues [54] point out that the empirical “research suggesting that SWB and eudaimonia are independent factors stems from three data sources: factor analytic studies, dependent correlations between narrow-band indicators or each type of well-being with a common outcome variable, and person-centered studies comparing different groups of people” (pp. 224–225). But our MDS study provides a new kind of support for distinguishing between eudaimonic and hedonic well-being.

### Dimension 2: The existential relatedness versus Epicurean independence dimension

Like Dimension 1, the existential relatedness versus Epicurean independence dimension (Dimension 2; the vertical dimension) comes through clearly in Figs 1 and 2. Quite unlike Dimension 1, Dimension 2 is a novel discovery in well-being research. Existential relatedness is an existing concept in well-being studies [55], but Epicurean independence is novel, as is the emergence of a dimension of well-being with existential relatedness at one end and Epicurean independence at the other. While the existential relatedness versus Epicurean independence dimension is original, it is not completely unexpected. Notions highly similar to both of these concepts were recently found to be highly salient in lay definitions of happiness in a study of 12 nations [56].

The variables positive relations with others, personal growth, purpose in life, and life satisfaction constitute the existential relatedness pole of Dimension 2. Based on Friedrich Schleiermacher’s notion of ‘*ein unmittelbares existentialverhältnis*’ [57], existential relatedness is the characteristic of being meaningfully interconnected with things other than oneself. This meaningful interconnectedness can include both spiritual and secular aspects, such as a profound connection with God, nature, or a group of people. Citing [58], Ai and colleagues [55] describe existential relatedness as a higher-order intrapersonal relatedness; “a deep feeling of connections with divine, humanity, the world, and the universe, each viewed as a whole” (p. 370). Separate bodies of literature describe concepts related to self-transcendence in a similar way [59, 60, 61].

The clustering of the existential relatedness variables on one side of Dimension 2 suggests that having deep connections with others, feelings of personal growth over time, having a meaningful purpose in life, and being satisfied with life tend to co-vary together, and in ways that the Epicurean independence variables do not. The covariance of these variables fits well with what is known about existential relatedness. For example, existential relatedness, be it from recognition of religious or secular interconnectedness, tends to promote “profound spiritual feelings [that] can permeate one’s entire life, operating as a powerful affective stimulus toward human existence and growth” (p. 370) [55], and brings many opportunities for feelings of meaning and purpose in life [62].

Existential relatedness is not the same concept as positive relations with others [55], but the connection between them is strong. A profound sense of one’s deep interconnectivity with people and things outside of oneself tends to encourage recognition of the value of other people and, thereby, regular tending to relationships. And, of course, well-tended relationships are more likely to be positive relationships. A strong sense of interconnectivity and the value of people and things outside of oneself might also lead one to be more satisfied with life, perhaps especially using a domain-based measure of life satisfaction as we do here. Reminiscent of Buddhist teachings, appreciating the interconnectivity and value of all things might lead to a reduction in the sense of being in competition with others, and feeling dissatisfied because of not having as many of the accoutrements of modern commercial life as one’s neighbors. This may explain why Mikulincer and Florian [63] found existential relatedness to be a resilience factor throughout the lifespan.

At the other end of Dimension 2, lies the Epicurean independence pole, which is constituted by autonomy and absence of negative affect. Named for Epicurus of Samos (c.341–271 BCE), Epicurean independence is achieved by being relatively free of painful experiences and not feeling the need to have ones’ views accepted by anyone but oneself. Being self-determining and independent in a way that enables one to resist social pressures to think and act in certain ways and to regulate one’s own behavior from within tends to be facilitated by a kind of self-directed detachment from everything. Epicurus (aligned with the Stoics on this issue) would point out that if one puts little to no emphasis on things outside of one’s control, then one is much less likely to be hurt by them [41]. For example, if one were to strongly desire to be liked by others, then one’s well-being is to a greater extent at the mercy of other people, decreasing one’s control over one’s own well-being, and increasing the chance of suffering. In this way, the detachment from things outside of oneself acts as a kind of shield against the vicissitudes of life, a buffer against the worst and most common kinds of suffering. As a result, independence and absence of negative affect can rise and fall together.

Epicurus advised others to seek a certain degree of independence from society, culture, tradition, and contemporary religious views. For example, contrary to the dominant beliefs at the time, Epicurus advised others to stop living in fear of, and attempting to appease, the Gods because they were very unlikely to be interested in what a mere mortal was up to [41]. Epicurus advocated for independence and mental and emotional self-reliance for the purpose of avoiding suffering. Indeed, avoiding suffering was the most important endeavor for Epicurus [64]. Epicurus viewed the absence of suffering (especially psychological pains, i.e., negative affect) as the ideal state, often referring to it as pleasure [65]. So, Epicurus would praise Epicurean independence, greatly appreciating the coincidence of self-reliance, free-thinking, and the absence of negative affect.

Considering the opposing poles together helps to further clarify the structure of Dimension 2. Understood in opposition to existential relatedness, Epicurean independence involves diminishing the significance of one’s interrelations with other people and things, especially any bonds of dependence. Ai and colleagues [55] have argued that existential relatedness involves more than just wanting to be cared for by other people, it extends to one’s desires to care *for* others as well, since this fully realizes the back-and-forth of true interrelationships and makes oneself an important part of the whole. In contrast, Epicurean independence involves mental self-sufficiency, a kind of self-directed detachment from everything, a stance that eschews the bonds of dependence as much as possible. Epicurus would admonish anyone that allowed their desires to reach out beyond their personal sphere of control, since this puts the individual at risk of suffering disappointment [65]. To a certain extent then, existential relatedness involves seeing oneself as a part of a whole with other people and other things, caring deeply for them, and perhaps even being vulnerable to them, in a way that Epicurean independence does not. In this way, existential relatedness encourages deep bonds, and Epicurean independence encourages control and power. The deep bonds of existential relatedness can result in great suffering. In times of injustice, or when a beloved community turns its back, one suffers more anguish as one is more attached to the relevant relationships. So it seems that, as a result of the suffering that results from deep caring for other people and things, existential relatedness doesn’t co-vary with absence of negative affect nearly as strongly as Epicurean independence does.

There is another potential reason for why existential relatedness doesn’t co-vary with absence of negative affect nearly as strongly as Epicurean independence; while Epicurus thought pain to be the greatest evil [65], many existential thinkers view the whole range of human experience, including negative emotions, to be integral to a meaningful human life [62]. So, belief in the interconnectedness of everything in relation to meaning in life might make the embracing of the whole range of emotions more likely. This stands in contrast to belief in the notion that negative emotions can and should be minimized at all costs, which probably leads to experiencing less negative affect.

### Expanding and supplementing factor analytic findings

The variables of the present study have typically formed two separate yet related factors in previous exploratory and confirmatory factor analytic studies: hedonic and eudaimonic well-being [9, 66, 67]. The present MDS results converge with the factor analytic results in showing that the hedonic and eudaimonic variables occupied two separate regions of the plots. Therefore, both factor analysis and MDS confirm the empirical distinction between hedonic and eudaimonic well-being. However, MDS provides additional insights that can supplement the factor analytic findings. Most importantly, by identifying a second dimension, MDS provides a more nuanced and detailed understanding of the underlying variable structure and pairwise relationships. Some of these novel insights are highlighted below.

Identifying the second dimension (i.e., existential relatedness versus Epicurean independence) is itself an important addition to the study of mental well-being. Yet, it also promotes a richer understanding of the first dimension. For example, although negative affect, a hedonic variable, can be distinguished from the eudaimonic variable autonomy, the two variables group together to form a concept that we labeled Epicurean independence. As such, both hedonic and eudaimonic variables can be further described based on their proximity to the poles of the second dimension. Therefore, identifying the second dimension helps in describing heterogeneity in the variables that define the first dimension. Similarly, identifying the first dimension helps in describing heterogeneity in the variables that define the second dimension.

As expected, in addition to uncovering the major dimensions in the data, and the position of every individual variable in each dimension, the MDS mapping of the data provides information on the one-by-one relationships between all variables under study in a way that is more intuitive than that provided by correlation matrices. Focusing on the national MDS plots in Fig 1, for example, it is clear that autonomy and life satisfaction are relatively far apart from each other across the MDS solutions, indicating a relatively weak association. The same goes for autonomy and positive relations. In contrast, personal growth and purpose in life are relatively close to each other. Self-acceptance and environmental mastery are even more closely related.

The MDS mapping of the data also allows for easy recognition of one-to-many relationships between the variables. For example, among the nine variables, autonomy is farthest apart from the rest of the variables, reflecting the weakest association with other variables. In contrast, environmental mastery and self-acceptance both lie somewhere in the central regions of the point configurations, reflecting that they demonstrate more or less equal correlations with all of the other variables. Due to their central position in the planes, they can be seen as central components of mental well-being, on both of the dimensions. However, given their distance from the dimension ends, they play a relatively reduced role in the interpretation of the dimensions. In all of the MDS plots presented, autonomy is the most isolated variable. This means that, in the context of the nine variables under study, autonomy is the least related to the others. As is often the case with isolated variables, autonomy also tends to fall a long way from the center of the variables. Autonomy’s position on the periphery of the MDS plots suggests that it is not as central to mental well-being as the other variables.

The present MDS results also permit the examination of cross-cultural differences in the structure of well-being. The results for Dimension 2 are fairly robust, again occurring in all of the national and gender groups in the study. The ubiquity of the result in our samples, and the great diversity of our samples, especially in terms of religiosity and individuality, gives reason to think that the existential relatedness versus Epicurean independence dimension is a broadly cross-cultural feature of well-being. Nevertheless, one variable, positive affect, occupies different places on Dimension 2 in the groups under study. In the national samples, positive affect falls within the existential connectedness cluster in the Japanese data, but closer to the Epicurean independence cluster in the data for the United States and Iran. Analysis of the MDS plots for the nation-gender subsamples, reveals that positive affect falls within the existential connectedness cluster in the data for Iranian males and for Japanese males and females. These results suggest that in collectivistic cultures such as Iran and Japan, positive affect is more likely to be aligned with positive relational experiences, whereas in individualistic cultures, positive affect is more likely to co-occur with the experience of autonomy.

This finding is in keeping with prior findings from cultural psychology. According to Kitayama, Duffy, and Uchida [68], in individualistic cultures, “happiness is typically construed as a personal achievement. Thus, individuals strive to attain happiness and, once obtained, happiness affirms the worth of the internal, private self. In contrast, … happiness in Japan is more socially anchored. It is seen as a realization of social harmony or state of mutual sympathy and understanding.” (p. 150). Therefore, self-attribution of positive/happy feelings is more likely to co-occur with autonomy and independence in individualistic cultures. In contrast, in collectivistic cultures where interpersonal harmony and adjustment of oneself to fit the group are emphasized, positive affect is more likely to be aligned with harmony with others and possibly the whole cosmos [61, 68]. The literatures on aversion to happiness [69] and ideal affect [70] also suggest that high arousal emotions (e.g., joy and excitement) are more strongly valued in individualistic cultures than in collectivistic cultures. One of the reasons for this is that high arousal emotions are perceived in collectivistic cultures as potential disrupters of interpersonal harmony [69, 71]. Therefore, this finding is consistent with the previous evidence about the cultural differences in the role and position of positive affect across cultures. Such novel insights confirm the great potential of MDS as a supplementary method in the study of mental well-being.

### Concluding remarks, implications, and limitations

Using a novel method, this study discovered a 2-dimensional structure in nine important mental well-being variables. Although the field of well-being has largely focused on the hedonic-eudaimonic dimension when describing the structure of mental well-being, as Belzak and colleagues [59] emphasize, the hedonic-eudaimonic dimension does not fully capture all existing variation in well-being concepts. The exploratory MDS analysis conducted in the present study revealed a second dimension (existential relatedness versus Epicurean independence) in the underlying structure of well-being variables. Our second dimension corresponds with Belzak and colleagues’ suggestion about the ignored dimension. They argue for self-transcendence to be seen as an important component of well-being alongside the well-established eudaimonic and hedonic components. Self-transcendence is defined as when “human agency is enhanced through the epistemic revelation or illumination that occurs in transaction with agencies, influences, and traditions beyond (antecedent to, greater than) the conscious self” (p. 134) [59]. This understanding of self-transcendence is closely related to the concept of existential relatedness that emerged in the present study.

Our second dimension also resonates with the emphasis on the distinction between personal vs social or agency vs communion constructs in psychology [27, 72, 73]. Notably, an exploratory MDS on a short well-being scale using an American sample also yielded a social vs. personal dimension [27]. Therefore, the present and previous results collectively imply the necessity of a second dimension to supplement the hedonic-eudaimonic dimension and capture the degree to which well-being variables are personal or social. Without a second dimeson being recognized, the complete relational system formed by well-being variables is not fully understandable. As noted before, a more nuanced understanding of the hedonic-eudaimonic dimension can also result from considering an extra dimension. At any rate, these results illustrate the value of exploratory methods such as MDS in discovering structures and dimensions in empirical data, and thereby supplementing factor analytic findings.

In line with the expectations of exploratory research, we clarified our findings by embedding them in relevant literatures. Now that we have clarified the eudaimonic well-being versus hedonic well-being, and the existential relatedness versus Epicurean independence dimensions of mental well-being, this novel structure can be tested and used in further well-being research. This further research could investigate the extent to which this new model elucidates new understandings of the rich construct of mental well-being, in much the same way that the development of the Big Five personality traits enabled a host of fruitful research on personality and personality’s role in other domains of interest [74, 75]. For example, the 2-dimensional structure could have important ramifications for therapy. People high in Epicurean independence and low in eudaimonic well-being might respond very differently to interventions focused on social-embeddedness than someone high in existential relatedness and low in hedonic well-being.

Our findings also have important implications for interdisciplinary well-being research, and the well-being approach to public policymaking. Economists researching well-being, have tended to use a very limited range of mental well-being variables, often just one item on life satisfaction. And, as can be seen in the makeup of the 2008 Stiglitz-Sen-Fitoussi Commission, economists seem to have had a much greater impact on public policy than other well-being researchers. More recently, psychologists and some economists and policymakers have argued for a broader range of mental well-being measures [76–78]. These broader ranges of mental well-being questions tend to focus on ensuring a reasonable coverage of hedonic and eudaimonic well-being questions. A goal of many of these researchers is to have mental well-being questions added to national censuses. Unfortunately, a census is such a huge undertaking that national statistical offices try to keep the number of questions strictly limited. As a result of these limits, there may only be space for a few mental well-being questions. If that is the case, then it may be better to ask questions that enable both of the dimensions of mental well-being that we have discovered here to be assessed. For example, a question about autonomy, which feeds into two dimensions and doesn’t co-vary closely with other variables, may be more important than a question about self-acceptance, which co-varies very closely with environmental mastery.

A specific application of the 2-dimensional model of mental well-being to public policy could help with the fraught issue of accounting for religious, secular, and hard-to-classify aspects of higher purpose in life. If the public request that policymakers measure the important aspects of mental well-being, then a model that includes existential relatedness might help catch the people falling between traditional religious and secular views, like those with “new age” beliefs that now make up fairly large proportions of the citizenry in various locations [79].

This novel 2-dimensional structure of mental well-being might also have important implications for cross-cultural well-being research, although our findings were robust across three distinct cultures, and across alternate measures of our variables, future research should attempt to replicate the 2-dimensional structure of mental well-being with other methods and in other cultures. Further cross-cultural validation of this structure may result in a measurable conception of mental well-being that is more cross-culturally robust than existing models. For example, the existential relatedness pole of Dimension 2 might better capture the cross-cultural range of spiritual, secular, and religious aspects of higher meaning than other models.

A potential limitation of the study is that the Iranian sample is a convenience sample of university students, whereas the other samples are of middle-aged people. It is possible that an MDS analysis of middle-aged Iranians would have generated outputs with distinct dimensions. This would mean that young Iranian adults seem to share a 2-dimensional structure of mental well-being with middle-aged Japanese and middle-aged respondents from the USA, but the same is not true of middle-aged Iranians. A follow-up study comparing young with middle-aged and possibly older respondents within and across cultures would help resolve this issue and could produce valuable findings in its own right.

Another potential limitation of any MDS analysis (indeed, many kinds of statistical analysis) is that the resulting dimensions reflect the choice of input variables. There exist very many measures of mental well-being, and selecting different variables would likely have resulted in different dimensions. For this reason, we chose variables that feature strongly in a wide variety of work on mental well-being, for which cross-cultural data are readily available, and that cover the main theories of mental well-being in philosophy, psychology, and economics. Given the identification of the existential relatedness versus Epicurean independence dimension was post hoc, future MDS research on mental well-being might test the robustness of this dimension by including new variables that fit the conceptual descriptions of these new poles. Most important in this regard would be to include variables aligned with the concepts of elevated experience or self-transcendence, such as inspiration and awe [59, 60]. The investigation of well-being (more generally) could also be advanced by running a similar MDS analysis that also includes social variables, such as social integration, social contribution, and generalized trust.

Future research should also consider using alternative scales of the variables that we used here. Our results from the Japanese sample demonstrate very little difference between the MIDUS life satisfaction scale and Diener and colleague’s Satisfaction With Life Scale [36]. A standout case for this kind of investigation is autonomy, which is conceptualized in self-determination theory [80] slightly differently from Ryff’s conceptualization [24]. Thus, it is important for future research to replicate the present results with alternative scales of the variables developed within various theoretical orientations.

## Supporting information

S1 Dataset Iranian data. (SAV)
